# Exploring the Synergy Between HPTLC and HPLC-DAD for the Investigation of Wine-Making By-Products

**DOI:** 10.3390/molecules24193416

**Published:** 2019-09-20

**Authors:** Tatiana Bernardi, Olga Bortolini, Alessandro Massi, Gianni Sacchetti, Massimo Tacchini, Carmela De Risi

**Affiliations:** 1 Dipartimento di Scienze Chimiche e Farmaceutiche, Università di Ferrara, Via Luigi Borsari 46, I-44121 Ferrara, Italy; olga.bortolini@unife.it (O.B.); alessandro.massi@unife.it (A.M.); 2 Dipartimento di Scienze della Vita e Biotecnologie, Sezione di Botanica Applicata, Piazzale Luciano Chiappini 2, Malborghetto di Boara, I-44123 Ferrara, Italy; gianni.sacchetti@unife.it (G.S.); massimo.tacchini@unife.it (M.T.)

**Keywords:** HPLC-DAD, HPTLC, densitometry, grape, *Vitis vinifera*, flavonoids, winery by-products

## Abstract

Both environmental and economic issues are increasingly pushing for the revalorization of agri-food by-products, including those arising from wine industry. Wastes produced from wine-making processes are important sources of biologically active compounds, mainly phenolic acids and flavonoids, which could be re-used for several applications, for example as additive surrogates or new ingredients in foodstuffs and/or pharmaceuticals. Therefore, the development of methods aimed at isolating, characterizing and quantifying molecules present in winery by-products acquires considerable importance in view of their re-utilization on a large scale. In this connection, this study demonstrated that high-performance thin-layer chromatography (HPTLC) and high-performance liquid chromatography with diode array detection (HPLC-DAD) can operate in synergy for the investigation of pomace and seed materials arising from both white and red cultivars of *Vitis Vinifera*. By virtue of fingerprint profiling, mass spectrometry (MS) interfacing and band comparison method, HPTLC enabled detection and identification of phenolic acids, non-anthocyanic flavonoids and anthocyanins. On the contrary, only anthocyanins could be identified by HPLC-DAD, and their subsequent quantification showed that malvidin-3-*O*-glucoside (oenin) was the most abundant one. In parallel, HPTLC has allowed to detect and quantify proanthocyanidins (PAC), showing that only catechin was present in the test samples. Both quantitative analytical methods were validated in terms of linearity, detection and quantification limits and precision.

## 1. Introduction

Grapes are among the most cultivated fruits in the world, with the greatest production being located in Europe (Italy, Spain, France), China and the USA. On the basis of the data reported by the Food and Agriculture Organization Corporate Statistical Database, the estimated global grape production in 2017 was more than 74,000 metric tons, distributed mainly among China (16.8%), Italy (10.9%) and the USA (9.6%) [1]. *Vitis vinifera* (European grape), *Vitis labrusca* and *Vitis rotundifolia* (North American grapes) can be cited as examples of the main grape cultivars, and of those *Vitis vinifera* is the most widely grown for wine production [2,3,4].

With particular regard to wine processing, it is well known that this procedure produces large amounts of solid by-products (30% by weight of the starting material), the majority of which are discarded without first being treated, with serious economic and environmental consequences [5,6].

Typically, the most abundant grape waste is pomace, also known as grape marc, which consists of seeds, stalks and skins and accounts for two thirds of the solid by-products [7]. It is calculated that 1 kg of grape pomace is produced for every 6 L of wine [8], just like 0.3 kg of solid by-products are obtained for every kg of processed grape [5].

Grape wastes contain a number of bioactive compounds, especially polyphenols, such as phenolic acids (derivatives of hydroxycinnamic acid and hydroxybenzoic acid) and flavonoids (flavonols, flavan-3-ols, anthocyanins and proanthocyanidins) [9,10,11]. For instance, studies on by-products derived from *Vitis vinifera* showed that skins are rich in hydroxycinnamic acids, anthocyanins, flavonols and flavan-3-ols, and importantly, anthocyanin compounds are completely absent in white grape pomace [12,13,14,15]. Moreover, seeds mainly contain flavan-3-ols, in particular (+)-catechin and (−)-epicatechin, along with (−)-epicatechin-3-*O*-gallate and procyanidins or proanthocyanidins with a varying degree of polymerization [16,17,18,19].

Thanks to the biological properties of their components (e.g., antioxidant, antiinflammatory, antimicrobial, antibacterial) [9,11,20,21,22,23,24,25], grape wastes could be re-used for different applications, for example as additive surrogates or new ingredients in food and/or pharmaceutical products [23,26]. That is why research efforts are desirable to achieve isolation, characterization and possibly quantification of molecules present in wine-making by-products, in order to re-use them on a large scale.

It is a known fact that high-performance thin-layer chromatography (HPTLC) is a powerful analytical tool for the analysis of complex mixtures [27]. This technique offers several advantages compared with other methods [28], namely, inter alia rapidity, simplicity, efficiency, cost-effectiveness, high sensitivity and reproducibility. Furthermore, typical HPTLC analysis requires a minimal sample preparation (dilution) and integrated automated systems, such as Automated Multiple Development (AMD) and Overpressured Layer Chromatography (OPLC), ensure additional advantages [29,30]. Last, but not least, the HPTLC method allows the simultaneous investigation of several samples in terms of comparison, identification and quantification [31,32], also by hyphenation with mass spectrometry (MS) [33].

HPTLC has been widely applied for the analysis of phenolic and flavonoid compounds contained in natural matrices [34,35,36,37,38], sometimes in combination with high-performance liquid chromatography (HPLC) [39,40,41,42]. In most cases, HPLC has been merely used to confirm the results obtained by HPTLC and vice versa, and no further information was deducted by making a comparison between the results of each technique, to the best of our knowledge.

Building on these literature data and our previous studies on the use of HPTLC for the inspection of complex mixtures [43,44,45,46,47,48,49], this work was meant to see whether HPTLC could work in synergy with HPLC-diode array detection (DAD) for the determination, separation and quantification of compounds from winery wastes.

This study was part of a project designed to revalorize wine-making by-products (pomace and desiccated seeds) obtained from vinification of Italian white (Trebbiano) and red (Lambrusco) cultivars of *Vitis Vinifera* in a wine-producing holding located in the municipality of Faenza (Emilia Romagna region, Italy). Once received, the winery materials were subjected to known extraction procedures to obtain suitable samples for subsequent HPTLC/HPLC-DAD analyses [25].

It is important to say that this work was carried out following specific directives of the company we have been collaborating with. In accordance to this, attention has been exclusively focused on the determination of polar constituents of grape wastes, while analysis of non-polar compounds was totally excluded.

Herein, we report the details of our studies which led us to demonstrate the complementarity between HPTLC and HPLC-DAD in both determination and quantification of flavonoid and non-flavonoid compounds within wine-making by-products. In doing so, we could exploit the advantages of each technique to push the boundaries of the other.

## 2. Results and Discussion

After obtaining the winery waste materials, initial attention was paid to obtain test samples for the subsequent analytical investigations.

In this respect, solid-liquid extraction seemed the most appropriate choice for our purposes, using pure ethanol-water combinations as suitable extraction solvents. In fact, it has been known that water, ethanol and their mixtures are the elected solvents for natural compounds. More precisely, 50% ethanol-water is well recognized as the best system for the extraction of phenolic derivatives from grape pomace [50].

In detail, 11 sample extracts were obtained from grape pomace and desiccated seeds by extracting with pure ethanol-water mixtures with increasing water content up to 100% [25].

**Figure 1:**
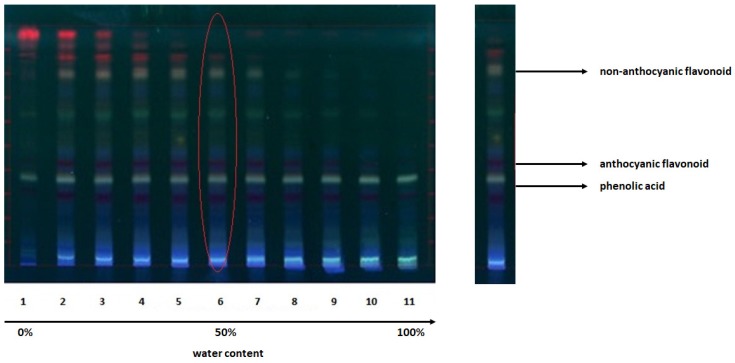
Typical image of high-performance thin-layer chromatography (HPTLC) separations for diagnostic fingerprint of sample extracts, after NP/PEG 400 derivatization.

Next, HPTLC fingerprint evaluation showed that the track with the most intense analyte spots corresponded to the samples obtained with 50/50 pure ethanol-water extraction solvent (Figure 1). Gratifyingly, this result was fully consistent with the literature data [50], and importantly, it was observed regardless of both wine-making by-product and extraction procedure.

At the same time, it has been possible to identify the major classes of organic compounds present in the extracts on the basis of analyte spots color, in turn resulting from derivatization with NP/PEG 400, that is 2-aminoethyl diphenylborinate (natural product reagent A, NP) and polyethylene glycol 400 (PEG 400). According to literature, orange-yellow spots were attributed to non-anthocyanic flavonoids, while phenolic acids and anthocyanins appeared as blue fluorescent and purple spots, respectively (Figure 1) [51].

At this stage, we tried to figure out what type of molecules was contained within the extracts. To this effect, two approaches were used, namely the HPTLC-MS method and direct comparison of bands with those of accessible standards in terms of both color and retardation factor (R_f_).

**Figure 2:**
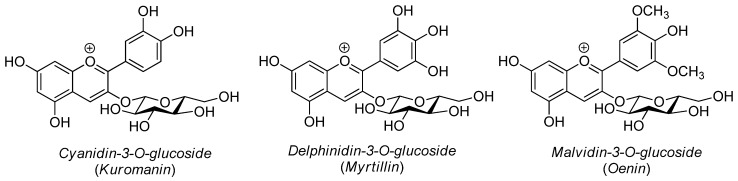
Chemical structures of the main anthocyanins identified by HPTLC-MS method.

In the first case, the only compounds detected were anthocyanins (Figure 2). These mainly include cyanidin-3-*O*-glucoside (kuromanin, *m/z* 449.388), delphinidin-3-*O*-glucoside (myrtillin, *m/z* 465.387) and malvidin-3-*O*-glucoside (oenin, *m/z* 493.441). In particular, malvidin 3-*O*-glucoside was present in greater proportion.

By contrast, chromatogram zones corresponding to phenolic acids and non-anthocyanic flavonoids did not produce any signal in the mass spectra. This was supposed to depend on their low concentration in the extracts, in line with recent literature data indicating that concentration levels higher than 250 ng/zone are needed for a valid visualization with MS interfaced to HPTLC [52].

**Figure 3:**
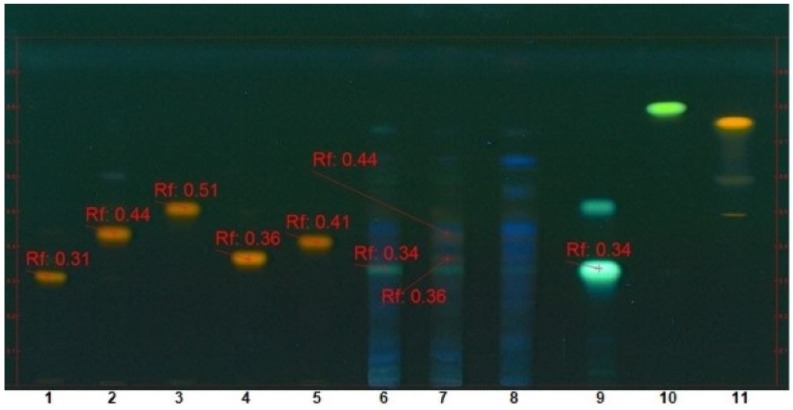
Identification of flavonoid and non-flavonoid components in extracts by band comparison method (1: quercetin 3-*O*-rutinoside, 2: quercetin 3-*O*-glucoside, 3: quercetin 3-*O*-rhamnoside, 4: quercetin 3-*O*-glucuronide, 5: quercetin 3-*O*-galactoside, 6: extract from red grape pomace, 7: extract from white grape pomace, 8: extract from red grape seeds, 9: caftaric acid, 10: kaempferol, 11: quercetin).

**Figure 4:**
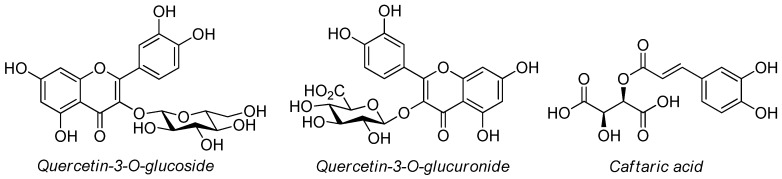
Chemical structures of flavonoid and non-flavonoid compounds identified by HPTLC fingerprint method.

The band comparison method led us to identify both non-anthocyanic flavonoid (i.e., quercetin 3-*O*-glucoside and quercetin 3-*O*-glucuronide, orange-yellow spots) and phenolic acid (i.e., caftaric acid, blue fluorescent spot) compounds (Figure 3 and Figure 4).

Building on these observations, we next looked at suitable analytical procedures for quantification of the detected compounds. For this task, the conventional HPLC-DAD technique seemed ideal, since DAD allows to analyze and monitor various types of molecules for each injected sample solution at one time, over a wide range of wavelengths.

Accordingly, extracts obtained under the optimized extraction conditions (50% pure ethanol in water) were filtered (membrane filter: 0.45 μm pore size) to preserve column efficiency and screened in a spectral range (220–550 nm) over which we expected absorption of the main classes of compounds that can be found in grape and its by-products. Known examples are gallic acid, catechin, epicatechin and *cis*-resveratrol (271–278 nm), *trans*-resveratrol (305 nm), *p*-coumaric acid (308 nm), caffeic, ferulic, synaptic and chlorogenic acids (323 nm) and even quercetin and rutin (365 nm). Other than that, a baseline drift at 280 nm can be assigned to proanthocyanidins (PAC), while absorption over the 500–535 nm range is typical of most of anthocyanins [53].

**Figure 5:**
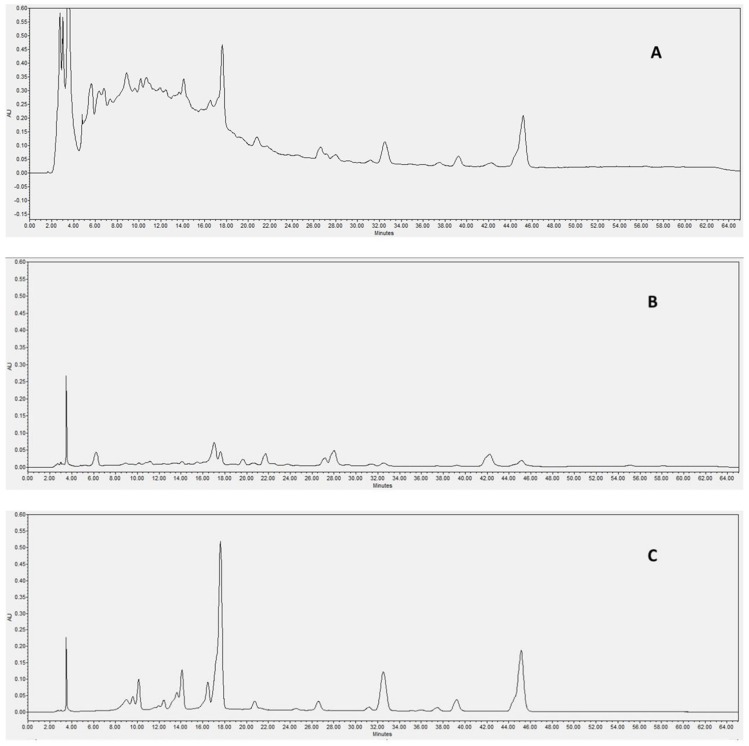
High-performance liquid chromatography with diode array detection (HPLC-DAD) chromatograms of a red grape pomace extract at (**A**) 280 nm, (**B**) 350 nm, (**C**) 520 nm.

One case in point is depicted in Figure 5. This shows the chromatograms recorded for a red grape pomace extract at three different wavelengths (280, 350, 520 nm) at which we would have expected absorption of phenolic acids, non-anthocyanic flavonoids and anthocyanins, respectively. The only successful result was observed at 520 nm, which indicated the presence of anthocyanin compounds.

Much to our surprise, anthocyanins were also found in the extracts deriving from seeds contained in red grape pomace samples. This result would appear to contradict known data showing that grape seeds do not contain these molecules [19]. However, it may be assumed that the direct contact of seeds with skins during the grape processing phases may produce this effect.

The absence of relevant peaks attributable to both phenolic acids and non-anthocyanic flavonoids in the test samples has been corroborated by HPLC-DAD analysis in the wavelength range 250–370 nm. This achievement was further supported by comparison with a set of retention time (t_r_) values of standards. In addition to this, the observed outcomes proved to be independent of sample concentration, as both types of derivatives could not be detected while concentrating the injected sample solutions to the very limits of filterability (up to 70 mg/mL).

Considering the above, the findings obtained by HPLC-DAD appeared to be in contradiction with those previously observed in HPTLC fingerprint profiles (Figure 1 and Figure 3). Nevertheless, we could offer some possible explanations. Most probably, the concentration of phenolic acids or non-anthocyanic flavonoids, even when present, is so low that they may be lost during sample pre-treatment (filtration) before HPLC-DAD analysis. It is worth mentioning that the supposed low concentration of these species matches the results observed in HPTLC-MS studies. Additionally, it is not excluded that the LOD of DAD was responsible for the incomplete (false) analytical evaluation.

**Figure 6:**
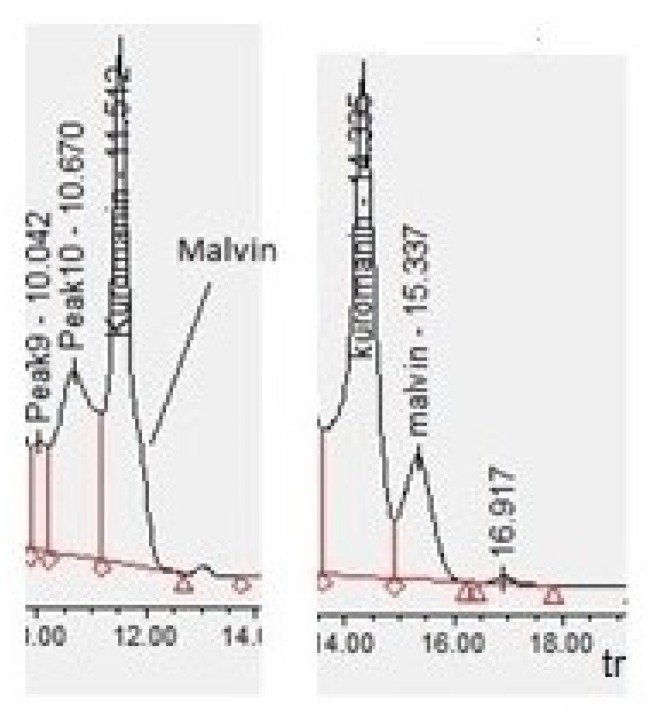
HPLC-DAD chromatographic separation of malvin and kuromanin (**left**: conventional Flamini and Favretto method; **right**: modified Flamini and Favretto method).

In order to unambiguously identify the anthocyanin species in the extracts, standard solutions (100 µg/mL) in HCl-methanol (0.5% *v/v*) were injected separately for t_r_ identification. In this regard, it can be said that the chromatographic conditions reported by Flamini and Favretto [54] had to be suitably modified to be adapted to the column system used (Luna^®^ C_18_), as detailed in Section 3.5. This was necessary because two of the standards, malvidin-3,5-*O*-diglucoside (malvin) and cyanidin-3-*O*-glucoside (kuromanin), had very similar t_r_ values (Figure 6, left). Under the changed conditions, it was possible to put one minute distance between the two substances, hence, distinguishing among them (Figure 6, right).

In doing so, delphinidin-3-*O*-glucoside (myrtillin, t_r_ = 11.62 min), cyanidin-3-*O*-glucoside (kuromanin, t_r_ = 14.33 min) and malvidin-3-*O*-glucoside (oenin, t_r_ = 22.40 min) were identified in the extracts, confirming what we found in HPTLC-MS investigations.

At a later stage, accurate quantification of anthocyanin compounds has been achieved by a calibration curve calculation method. For this purpose, four working standard solutions with concentrations of 10, 40, 70 and 100 μg/mL, prepared from stock standard solutions (1 mg/mL concentration), were injected as mixtures (at the same level of concentration), in triplicate. The compound peak areas were calculated for each concentration level and a curve was plotted (compound concentration against peak area). The calibration curves were checked daily to verify that peak areas were in the calibration range; otherwise, the calibration plots were recalculated in full.

**Figure 7:**
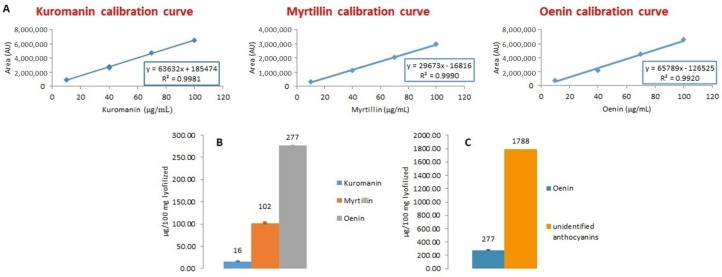
(**A**) Calibration curves of kuromanin, myrtillin and oenin with their corresponding regression equations and coefficients of determination; (**B**) quantification of kuromanin, myrtillin and oenin; (**C**) quantification of unidentified anthocyanins.

Very good correlations were observed, with R^2^ values between 0.9920 and 0.9990, indicating the linearity of the method at the working concentration range (10–100 μg/mL) (Figure 7A). Final quantification showed that oenin was the most abundant anthocyanin, followed by myrtillin and kuromanin in that order (Figure 7B).

Unfortunately, it was not possible to associate all the peaks in the chromatograms to the standard compounds at our disposal. In such cases, we quantified the content of unidentified anthocyanins based on the calibration curve of oenin, according to known directions (Figure 7C) [55].

Additionally, the sensitivity of the HPLC-DAD method was evaluated in terms of limit of detection (LOD) and limit of quantification (LOQ) (Table 1), and precision was around 0.8% in all cases.

It must also be said that a baseline drift at 280 nm provided an important clue to the presence of PAC. It is a very well-known fact that analysis of these derivatives is possible after thiolysis, as indicated in the literature [56]. In relation to this issue, we carried out preliminary tests, however, regrettably, thus far it has not been possible to achieve any satisfactory results.

Nonetheless, we saw great potential in the HPTLC technique for identification and quantification of PAC. On this subject, it is well established that when we talk of the latter, we generally refer to the monomer precursors catechin and epicatechin, as also to species with higher molecular weight, especially epigallocatechin and epigallocatechin gallate.

**Figure 8:**
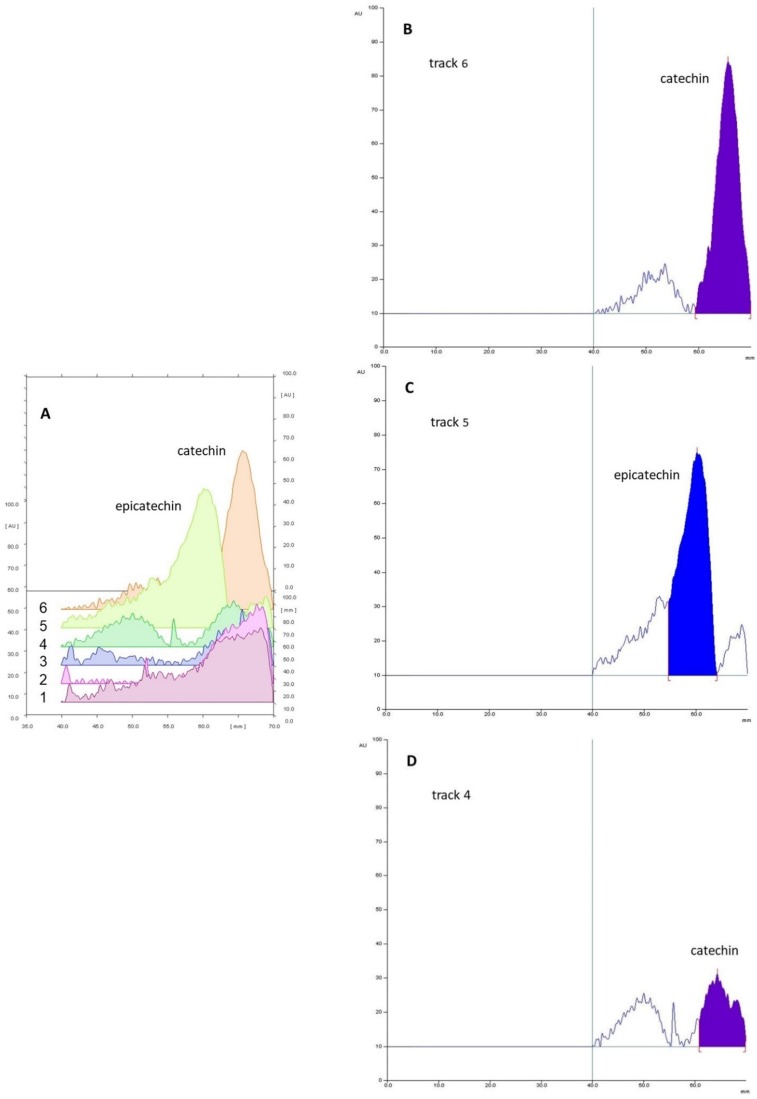
(**A**) Densitograms of four representative extracts (tracks 1–4), epicatechin standard solution (track 5) and catechin standard solution (track 6); (**B**) detail of standard catechin densitogram; (**C**) detail of standard epicatechin densitogram; (**D**) illustrative densitogram of sample extracts showing the complete lack of epicatechin.

Based on this, a preliminary experiment was run to verify the presence of either catechin or epicatechin (or both) in extracts, under the protocol reported by Vovk et al. [57]. Thus, two standard solutions of catechin and epicatechin (120 μg/mL) were compared with four representative extracts deriving from red (white) grape pomace and seed materials. Elution (n-propanol/water/acetic acid 4/2/1 *v/v/v*), derivatization with vanillin-phosphoric acid reagent (VPA) and final registration of the corresponding densitograms (540 nm) demonstrated that epicatechin was not present at all in our samples (Figure 8). It is important to highlight that densitograms obtained from the sample extracts showed pretty poor peaks. This was likely due to a low catechin content producing weak and therefore less defined signals.

Moreover, we have to say that VPA was chosen as the derivatizing agent in order to exclusively reveal the molecules in question. This strategy was thought to tackle the possible multiple spot revelation due to band overlapping. In fact, VPA is able to discriminate a single molecule class in contrast to NP/PEG 400, which is better suited to define the composition of a complex matrix in its entirety.

The next stage was to discriminate the catechin monomer from its higher molecular weight derivatives epigallocatechin and epigallocatechin gallate. To this end, we adopted a reported HPTLC separation method validated for demonstrating adulteration of grape seed extracts [58].

**Figure 9:**
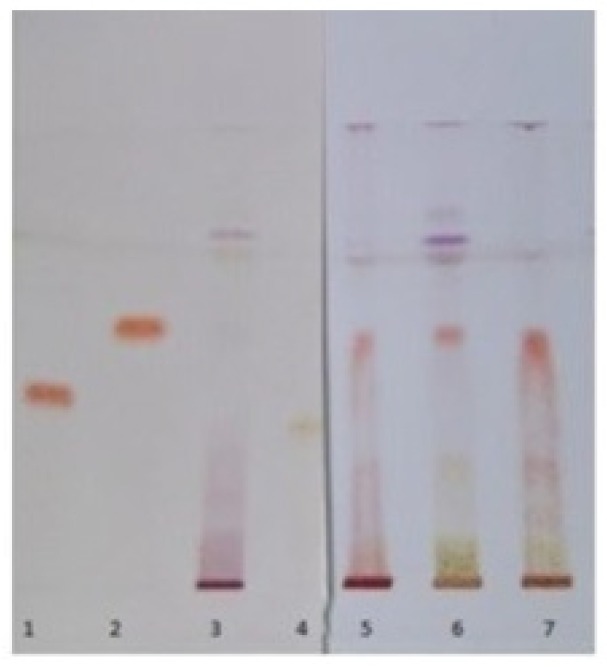
HPTLC elution of the extracts next to catechin, epigallocatechin and epigallocatechin gallate standards (1: epigallocatechin, 2: catechin, 3: red grape pomace extract, 4: epigallocatechin gallate, 5: red grape seed extract, 6: white grape pomace extract, 7: white grape seed extract).

In detail, catechin, epigallocatechin and epigallocatechin gallate standards (see Section 3.4) were deposited onto the HPTLC plate together with all the above extracts. After elution (toluene/acetone/formic acid 4.5/4.5/1.5 *v/v/v*) and derivatization (VPA), it has been found that catechin was present as the sole compound (Figure 9).

Then, quantification of catechin was run via its calibration curve between 240 and 420 μg/mL. For the construction of the latter, the working standard solutions (4, 5, 6 and 7 μL) have been spotted in three replicates. After elution (toluene/acetone/formic acid 4.5/4.5/1.5 *v/v/v*), derivatization (VPA) and densitogram registration (540 nm), the peak area of each compound has been calculated (for each concentration value) and a calibration plot of peak area versus concentration was drawn.

It is worth noting that a proper selection of both chromatographic system and derivatization agent is fundamental to overcome the difficulties concerning HPTLC quantification in a complex mixture. In our hands, the chosen chromatographic conditions and VPA derivatization have enabled us to detect catechin exclusively. This gave us the chance to smoothly perform its quantification.

**Figure 10:**
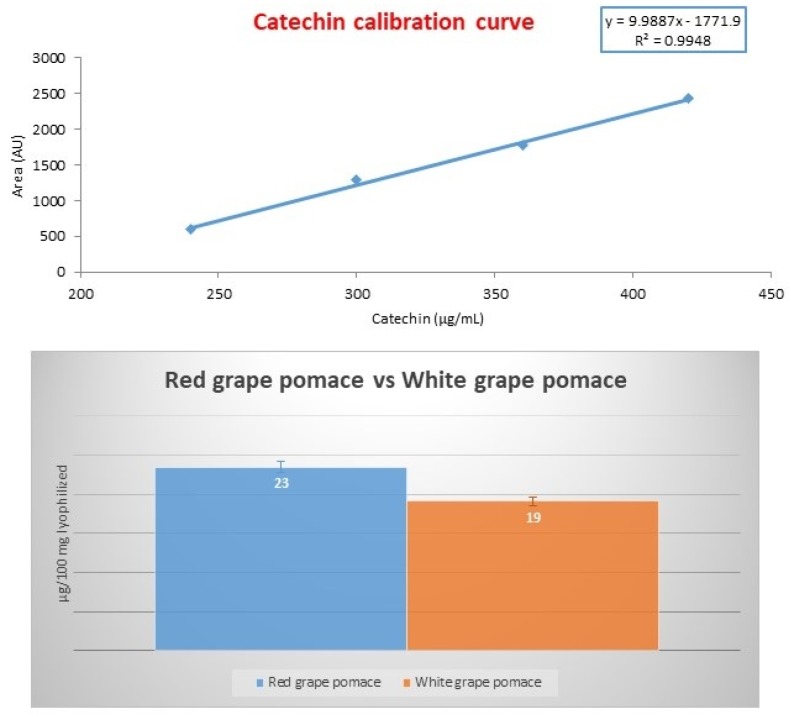
(**A**) Calibration curve of catechin for sample extracts derived from red and white grape pomace; (**B**) comparison of catechin content in red and white grape pomace extracts.

A representative case is reported in Figure 10 for red and white grape pomace extracts. A linear relationship exists within the test range (R^2^ = 0.9948). Quantification data revealed that the samples selected do not differ much in catechin content. LOD and LOQ values were 22.12 and 55.05 μg/mL, respectively, and precision was 0.3%.

## 3. Materials and Methods

### 3.1. Reagents and Apparatus

HPLC grade ethyl acetate, methyl alcohol, acetic acid (99%), formic acid (98–100%) and toluene as well as analytical grade hydrochloric acid (37%), phosphoric acid (85%), ethyl alcohol and dichloromethane were purchased from Sigma-Aldrich (St. Louis, MO, USA) and Merck (Darmstadt, Germany). The anthocyanin standards (cyanidin-3,5-*O*-diglucoside, cyanidin-3-*O*-glucoside, delphinidin-3-*O*-glucoside, malvidin-3,5-*O*-diglucoside, malvidin-3-*O*-glucoside, delphinidin) and also (+)-catechin, (−)-epicatechin, (−)-epigallocatechin and (−)-epigallocatechin gallate were provided by Extrasynthese (Genay, Lyon area, France). The reagents for derivatization steps, namely 2-aminoethyl diphenylborinate (natural product reagent A, NP) and polyethylene glycol 400 (PEG 400), were obtained from Sigma Aldrich, while vanillin was bought from BDH (Poole, UK). Bidistilled water was used as solvent for each type of application method, i.e., extraction and sample preparation.

### 3.2. Waste Materials

Winery by-products have been provided by Caviro (Faenza, Emilia Romagna, Italy). They were of two types, pomace and desiccated seeds, arising from both white (Trebbiano) and red (Lambrusco) *Vitis vinifera* varieties. In particular, the pomace materials contained stalks, seeds and skins, which were recovered after completion of fermentation and ethanol recovery (via extraction/distillation) during the wine-making process.

In order to ensure preservation of all samples, we followed the recommendations of our provider. Thus, samples were dried to constant weight at 70 °C in a ventilated oven for an optimized 24 h time. In the case of pomace, this procedure proved mandatory to avoid fermentation, due to its high water content.

In preparation for the extraction step, the dried samples have been crushed with a variable-speed rotor mill (Pulverisette 14, Fritsch, Idar-Oberstein, Germany) using two sieves (0.5 mm mesh for pomace, 2.0 mm mesh for seeds), and then they have been stored at −18 °C.

### 3.3. Extraction Procedures

Sample extracts were obtained from the waste materials by solid-liquid extraction using water and pure ethanol as the extraction solvents. According to known directions (solvent/solid ratio: 13 mL/g of dried waste, extraction time: 80 min) [25], a Naviglio^®^ extractor (Atlas Filtri, Limena, Italy) [59] and an ultrasonic cleaning bath (Ultrasonik 104X, Ney Dental International, Bloomfield, NJ, USA) have been used for the purpose, with each extraction being performed in triplicate. After, ethanol was evaporated off and the residue was lyophilized to facilitate handling and storing.

### 3.4. HPTLC Analysis: Fingerprint, Qualitative and Quantitative Evaluations

For HPTLC fingerprint evaluation [51], the extract solutions (8 μL) were spotted on 20 × 10 cm plates (silica gel 60 F_254_, Merck Schweiz AG, Zug, Switzerland) as 6 mm wide bands (10 mm away from both bottom and left side) at a track distance of 11.3 mm. Double development was run in a twin-through chamber using two solvent mixtures, first eluent A (ethyl acetate/formic acid/acetic acid/water 100/11/11/20 *v/v/v/v*, 40 mm elution front) then eluent B (ethyl acetate/acetic acid/toluene 90/10/100 *v/v/v*, 80 mm elution front). In both cases, a 20 min time-limit for chamber pre-saturation was applied. After complete elution, the thin-layer was dried in a ventilated oven at a temperature of 30 °C for 10 min. The separation results were acquired at 366 nm (Hg lamp) by a TLC Visualizer (CAMAG, Muttenz, Switzerland) after chemical derivatization with NP/PEG 400. For the latter, the thin-layer was first dipped into a NP solution (1 g of 2-amminoethyl diphenylborinate in 200 mL of ethyl acetate) for 1 s at the speed of 1 cm/s. Then, the same layer was immersed in a PEG 400 solution (1 g of PEG 400 in 20 mL of dichloromethane) at the same speed and dwell time. After every immersion, the plate was dried for 1 min with warm air.

For mass spectrometry (MS) investigation, the compounds were analyzed in positive ion mode using an ion trap (Thermo LC-QDuo, Thermo-Finnigan, Waltham, MA, USA). Capillary source voltage was set at 10 V, source voltage was 4.57 kV, and capillary temperature has been set at 160 °C. Elution of bands was run with the HPTLC-MS Interface (CAMAG) using methanol at a flow rate of 0.1 mL/min.

For catechin/epicatechin discrimination, working standard solutions at a concentration of 60 mg/L were obtained from stock standard solutions (1000 mg/L) using 50/50 ethanol-water mixture as the solvent in either case. Furthermore, sample extract solutions in 50% ethanol-water were prepared over the range of concentrations of 20 to 70 mg/mL. The standard and extract solutions (2 μL and 20 μL, respectively) were applied to the thin-layer as 5 mm wide bands (track distance: 15 mm, deposition front: 10 mm from the bottom side and 10 mm from the left side). According to Vovk directions, a 10 × 10 cm cellulose plate (Merck Schweiz AG) and the mobile phase n-propanol/water/acetic acid (4/2/1 *v/v/v*) were used as the chromatographic system [57]. The thin-layer was developed up to 60 mm from the deposition line (without pre-saturation of the developing chamber), then, it was dried in a ventilated oven at 30 °C for 10 min. Finally, derivatization was effected using vanillin-phosphoric acid reagent (VPA, formed by 0.5 g of vanillin, 12.5 mL of ethanol, 12.5 mL of water and 35 mL of phosphoric acid).

With regard of the HPTLC separation, which allowed to distinguish catechin from its higher molecular weight compounds (i.e., epigallocatechin and epigallocatechin gallate), the chromatographic system relied on the utilization of HPTLC silica gel plates (size 20 × 10 cm, 60 F_254_, Merck Schweiz AG) and a solution of toluene/acetone/formic acid (4.5/4.5/1.5 *v/v/v*) as the mobile phase in unsaturated chamber [58]. Stock standard solutions and working solutions were prepared exactly as done for the aforementioned catechin/epicatechin separation. The deposited bands (6 mm wide, 12 mm apart) were developed to a height of 70 mm from the deposition front, and thereafter, derivatization has been carried out with VPA (dwell time: 1 s, immersion speed: 5 cm/s), followed by documentation under 540 nm light (tungsten lamp).

On the subject of catechin quantification, aliquots of 4, 5, 6 and 7 μL deriving from the catechin working solution (60 mg/L) have been used (5 mm wide bands, track distance: 15 mm, deposition front: 10 mm from the bottom side and 7 mm from the left side). Elution, derivatization and acquisition were performed as already indicated for the HPTLC separation of catechin, epigallocatechin and epigallocatechin gallate.

The polarity of stationary phases, mobile phase mixtures as well as time for chamber pre-saturation have been thoroughly optimized in order to effectively elute polar species and ensure the best resolution (separation).

In all cases, deposition was done with the Linomat V depositor (CAMAG, Muttenz, Switzerland) using a 100 μL syringe (dosage speed: 10 nL/s). This was cleaned three times with pure ethanol/water (50/50 *v/v*) and twice with the test sample solution before each deposition operation. Images and densitograms have been obtained by SCANNER 3 (CAMAG) using the software winCATS Planar Chromatography Manager, version 1.4.4.6337 (CAMAG).

### 3.5. HPLC-DAD Analysis: Qualitative and Quantitative Determinations

The analytes were detected using a HPLC-DAD apparatus (Waters Corporation, Milford, MA, USA) equipped with 1525 binary pumps, 2998 photodiode array detector and Breeze 2.0 software system for data acquisition and processing (version 6.20.00.00, Service Pack A, Waters Corporation, Milford, MA, USA).

The analyses were performed with a Luna^®^ RP C_18_ column (250 × 4.6 mm id, 5 μm, Phenomenex, Torrance, CA, USA) at 25 °C, in the linear wavelength range between 220 and 550 nm, with 20 μL injection volumes. The analytical method is a modification of the procedure described by Flamini and Favretto [54]. The eluent system is a binary mixture consisting of water/formic acid 90/10 *v/v* (solvent A) and methanol/water/formic acid 50/40/10 *v/v/v* (solvent B). The gradient used (flow rate: 1 mL/min) increases from 15% B to 45% B in 25 min, from 45% B to 70% B in 30 min, from 70% B to 90% B in 10 min, from 90% B to 99% B in 5 min, and finally from 99% B to 15% B in 5 min to re-equilibrate the column and restore the initial state of the gradient.

Anthocyanin stock standard solutions (1000 mg/L) were prepared in MeOH and stocked at −18 °C in either dark glass containers or vessels coated with aluminum foil to protect the flavonoid compounds from light-induced degradation [60,61]. Working solutions (10, 40, 70 and 100 µg/mL) were obtained in HCl-methanol solvent (0.5% *v/v*), which is appropriate for anthocyanins as it allows to preserve both color and stability over time, and used for quantification purposes.

Sample extract solutions (concentrations between 20 and 70 mg/mL) were prepared in 50% pure ethanol-water and analyzed on the same day due to chemical instability of the monitored compounds in solution.

### 3.6. Validation of the Methods

Both quantitative HPTLC and HPLC-DAD methods have been validated according to the ICH guidelines in terms of linearity, LOD, LOQ and precision [62].

Linearity was assessed through calibration curves, in turn obtained by plotting the response factor (chromatographic peak area) against analyte concentration for a set of working standard solutions over a certain concentration range. Analyses were performed in triplicate and for each calibration plot both the coefficient of determination (R^2^) and the linear regression equation have been calculated and provided to prove linearity.

Speaking of LOD and LOQ, their calculation was different depending on the method of analysis. For quantitative HPLC-DAD, detection and quantification limit values were determined on the basis of signal-to-noise (S/N) ratio. In accordance with this, LOD and LOQ were 3 and 10 times the value of the noise level, respectively. In the case of the HPTLC-densitometric method, LOD and LOQ have been obtained relying on the standard deviation of intercept and the slope of the calibration curve. In this latter case, the computation was made by means of general Equation (1):

(1) $$\mathrm{LOD}/\mathrm{LOQ}=F\times \frac{SD}{S}$$

where F is 3.3 for LOD and 10 for LOQ, SD represents the standard deviation of y-intercept, and S is the slope of the regression plot.

In relation to precision, this was determined as the percent standard deviation (% SD) of three parallel analyses conducted on the same established sample.

## 4. Conclusions

In conclusion, this study has demonstrated that two well established analytical tools, namely HPTLC and HPLC-DAD, may be used in synergy to investigate complex natural mixtures, such as wine-making by-products. These techniques allowed us to identify and quantify polyphenol compounds in pomace and seed materials, in turn derived from white and red grape (*Vitis Vinifera*) varieties. What seems to emerge from the results achieved is the possibility to exploit the benefits of one technique to compensate for the limits of the other. Thus, typical features of HPTLC (minimal sample manipulation, i.e., dilution, low limit of detection, multiple derivatization) have likely helped achieve a greater extracts characterization (phenolic acids, non-anthocyanic flavonoids, anthocyanins) that supplemented the limited information given by HPLC-DAD (anthocyanins) for the same sample types. Although HPLC-DAD has the advantage of being able to monitor the content of the matrices in typical wavelength ranges with just one injection, either the sample pre-treatment (filtration) or the LOD of the detector may provide incomplete (false) results, in apparent contradiction with the HPTLC response. This being the case, each technique is not just for confirming the data provided by the other, as usually done, but each of them adds to the other. We are aware that some problems still remain both in the HPTLC method (e.g., band overlapping and robust quantification in a complex mixture) and in the HPLC approach (e.g., identification of phenolic acids and non-anthocyanic flavonoids as well as detection of PAC), but we trust that these preliminary results may represent a good basis for further development that is underway.

## Figures and Tables

**Table 1:** Limit of detection (LOD) and limit of quantification (LOQ) values for the HPLC-DAD method.^1^

Anthocyanin Compound	LOD^2^	LOQ^2^
Kuromanin	0.29	0.88
Myrtillin	0.54	1.63
Oenin	0.41	1.24

^1^ LOD and LOQ values as μg/mL. ^2^ LOD and LOQ values were calculated based on the signal-to-noise (S/N) ratio.
